# Comprehensive gene expression meta-analysis identifies signature genes that distinguish microglia from peripheral monocytes/macrophages in health and glioma

**DOI:** 10.1186/s40478-019-0665-y

**Published:** 2019-02-14

**Authors:** Verena Haage, Marcus Semtner, Ramon Oliveira Vidal, Daniel Perez Hernandez, Winnie W. Pong, Zhihong Chen, Dolores Hambardzumyan, Vincent Magrini, Amy Ly, Jason Walker, Elaine Mardis, Philipp Mertins, Sascha Sauer, Helmut Kettenmann, David H. Gutmann

**Affiliations:** 1 Max Delbrück Center for Molecular Medicine in the Helmholtz Association, Berlin, Germany; 2 Department of Neurology, Washington University School of Medicine, Box 8111, 660 S. Euclid Avenue, St. Louis, MO 63110 USA; 3 Department of Pediatrics, Emory University, Atlanta, GA USA; 4 McDonnell Genome Institute, Washington University School of Medicine, St. Louis, MO USA

**Keywords:** Microglia, Monocytes, Glioma, CNS; RNA sequencing, Microarray, Macrophages

## Abstract

Monocytes/macrophages have begun to emerge as key cellular modulators of brain homeostasis and central nervous system (CNS) disease. In the healthy brain, resident microglia are the predominant macrophage cell population; however, under conditions of blood-brain barrier leakage, peripheral monocytes/macrophages can infiltrate the brain and participate in CNS disease pathogenesis. Distinguishing these two populations is often challenging, owing to a paucity of universally accepted and reliable markers. To identify discriminatory marker sets for microglia and peripheral monocytes/macrophages, we employed a large meta-analytic approach using five published murine transcriptional datasets. Following hierarchical clustering, we filtered the top differentially expressed genes (DEGs) through a brain cell type-specific sequencing database, which led to the identification of eight microglia and eight peripheral monocyte/macrophage markers. We then validated their differential expression, leveraging a published single cell RNA sequencing dataset and quantitative RT-PCR using freshly isolated microglia and peripheral monocytes/macrophages from two different mouse strains. We further verified the translation of these DEGs at the protein level. As top microglia DEGs, we identified *P2ry12*, *Tmem119, Slc2a5* and *Fcrls*, whereas *Emilin2*, *Gda, Hp* and *Sell* emerged as the best DEGs for identifying peripheral monocytes/macrophages. Lastly, we evaluated their utility in discriminating monocyte/macrophage populations in the setting of brain pathology (glioma), and found that these DEG sets distinguished glioma-associated microglia from macrophages in both RCAS and GL261 mouse models of glioblastoma. Taken together, this unbiased bioinformatic approach facilitated the discovery of a robust set of microglia and peripheral monocyte/macrophage expression markers to discriminate these monocyte populations in both health and disease.

**Electronic supplementary material:**

The online version of this article (10.1186/s40478-019-0665-y) contains supplementary material, which is available to authorized users.

## Introduction

Microglia represent the major population of myeloid cells (monocytes) in the healthy brain parenchyma, where they perform vital functions, ranging from homeostatic surveillance to serving as the first line of immune defense [45]. Microglia originate from primitive macrophages that exit the yolk sac at mouse embryonic day 8.5, and subsequently colonize the neuroepithelium to become the resident CNS macrophage population [34]. Under certain pathological conditions, peripheral monocytes can enter the CNS from the blood through a disrupted blood brain barrier [13]. While there is little turnover of microglia in the healthy brain, blood monocytes/macrophages exhibit a high turnover rate [46]. In addition to their different origins, microglia and peripheral monocytes/macrophages have distinct functions in the setting of brain pathology. For example, opposing effects of microglia and infiltrated monocytes/macrophages have been reported in malignant brain tumors (glioblastoma) [5, 6, 9].

Defining the individual contributions of microglia and infiltrated monocytes/macrophages has been hampered by a lack of reliable markers that discriminate these two macrophage populations. First, while monocytes/macrophages are of haematopoetic origin, their transcriptome substantially overlaps with microglial gene expression [7, 16]. Second, some of the genes/proteins used to distinguish these two populations are not exclusively expressed by either microglia or macrophages, but are only relatively enriched. This includes the protein tyrosine phosphatase receptor type C (CD45), the fractalkine receptor (CX3CR1), and the C-C chemokine receptor type 2 (CCR2) [1, 4, 10, 15, 17, 25, 47]. Third, discriminatory genes frequently employed to identify peripheral monocytes/macrophages, such as CD45 or CCR2, can be induced in microglia associated with brain tumors (glioma). Similarly, blood-derived macrophages have been reported to decrease their *Ccr2* expression upon entry into the brain under pathological conditions, while these same conditions induce *Ccr2* expression in microglia [1, 4, 11, 32, 40, 47]. Lastly, while other monocyte population-specific markers have been identified, including TMEM119, it is not clear that they can reliably distinguish microglia from peripheral monocytes/macrophages in the normal brain and in the setting of CNS pathology [3, 5, 7, 14, 28].

In an effort to generate a resource for discriminating microglia from peripheral monocyte/macrophage markers in the normal brain and in the setting of disease, we employed a meta-analytic approach using five published mouse transcriptomal datasets, where profiles from both microglia and peripheral monocyte/macrophage populations were included. In combination with several secondary selection filters and proteomic validation, a robust set of microglia and monocyte/macrophage DEGs was identified and shown to discriminate microglia from monocyte/macrophages both in the normal brain and in the context of experimental murine glioma.

## Materials and methods

### Animals and ethics statement

All mice used for quantitative RT-PCR or proteomics validation were males, which were maintained on a C57BL/6J genetic background. Animals were handled according to governmental (LaGeSo) and internal (Max Delbrück Center for Molecular Medicine) rules and regulations. For quantitative RT-PCR validation, *Cx3cr1*^EGFP/WT^;*Ccr2*^RFP/WT^ mice were used to isolate microglia and peripheral monocyte/macrophages, respectively. Mice were kept in the animal facility using 12 h of light and dark cycle, with food and water ad libitum. All experiments were performed in strict accordance with the German Animal Protection Law as approved by the Regional Office for Health and Social Services in Berlin (Landesamt für Gesundheit und Soziales, Berlin, Germany, Permit Number (T0014/08, O360/09, A-0376/17). Adult mice were euthanized by intraperitoneal injection of pentobarbital (Narcoren, Merial GmbH, Hallbergmoos, Germany). All efforts were made to minimize pain and suffering.

Animals for the experimental glioma studies were housed in the Cleveland Clinic Biological Resource Unit or the Emory University Division of Animal Resources. All experimental procedures were approved by the Institutional Animal Care and Use Committee of the Cleveland Clinic (Animal Protocol 2013–1029; approved June 25, 2013) and Emory University (Protocol #2003253; approved September 15, 2015), and performed in strict accordance with the recommendations in the Guide for the Care and Use of Laboratory Animals of the National Institutes of Health. All surgeries were performed under anesthesia, and all efforts were made to minimize suffering.

### Experimental high-grade glioma mouse models

*Ntv-a*;*Ink4a-Arf−/−*;Gli-luc mice developed gliomas following intracranial RCAS-PDGFB injection by 6–8 weeks of age. Tumors were subsequently collected at 10–13 weeks of age [20]. Mice of both sexes were used in these experiments. Control mice were matched by genotype, gender, and age and did not receive RCAS injections.

### Fluorescence activated cell sorting (FACS) of microglia and spleen monocytes/macrophages

12–14-week-old male C57/BL6 mice were transcardially perfused under deep anesthesia with 1x Phosphate Buffered Saline (PBS). Brains were isolated, and after removal of the cerebellum and brainstem, dissociated into a single-cell suspension using Adult Brain Dissociation Kit (Miltenyi, Bergisch Gladbach, Germany) and the gentleMACS dissociator (Miltenyi), according to manufacturer instructions. Subsequently, cells were washed in PBS, passed through a 35 μm nylon mesh, counted and stained with anti-Mouse CD11b + PE-Cyanine7 (Life technologies | Thermo Fisher Scientific, Waltham, Massachusetts, USA) and anti-Mouse CD45 eFluor 450 (Life technologies | Thermo Fisher Scientific) for 20mins on ice. Spleens were manually dissociated in dissociation buffer (PBS containing 5.6% Glucose and 15 mM Hepes), and filtered through a 70 μm strainer and then passed through a 35 μm nylon mesh. Subsequently, the resulting single cell solution was centrifuged at 500 g for 5 min, and red blood cells were lysed for 10 min in ACK buffer at room temperature. PBS was added, samples centrifuged, and the supernatant was discarded prior to staining the cells with anti-Mouse CD11b^+^ PE-Cyanine7 (Life technologies) and anti-Mouse CD45 eFluor 450 (Life technologies), anti-Mouse Ly6G-FITC (eBioscience | Thermo Fisher Scientific, Waltham, Massachusetts, USA) and anti-Mouse Ly6C-PerCP/Cy5.5 (eBioscience | Thermo Fisher Scientific) for 20mins on ice. After staining, cells were washed once in PBS, and sorted on a FACS Aria flow cytometer (BD Biosciences, Franklin Lakes, USA) according to the specified gating strategy: microglia were sorted as CD11b^+^CD45^low^ cells, whereas spleen monocytes/macrophages were isolated as CD11b^+^CD45^high^Ly6G^low^Ly6C^high^ cells. For brain and spleen samples derived from *Cx3cr1*^GFP/WT^*;Ccr2*^RFP/WT^ mice, cells were collected after centrifugation, washed in PBS, and the cell pellets snap frozen for storage at − 80 °C.

### Fluorescence activated cell sorting (FACS) of glioma-associated microglia and monocytes/macrophages from RCAS tumor mice

Whole brains were collected from anesthetized and Ringer’s solution-perfused 3-month-old *Ntv-a;Ink4a-Arf*^−/−^;Gli-luc female and male mice and stored overnight in cold media. Tumors (*n* = 4), as well as age and gender matched forebrains from naïve animals (*n* = 4), were dissected and dissociated. Microglia and monocytes/macrophages were isolated using a Percoll density gradient for antibody-mediated flow sorting [8]. Forward Scatter (FSC) and Side Scatter (SSC) were used to determine viable cells, and appropriate controls were included for compensation and gating of stained populations (single, isotype and fluorescence minus one (FMO) controls) [2]. Two cell populations were collected from tumors: CD45^high^ (infiltrated monocytes/macrophages); CD45^low^ (microglia) cells that were also CD11b^+^, F11r^+^, Ly6G^neg^, Sell^neg^, CD3^neg^, CD19^neg^, and NK1.1^neg^ cells. One population was collected from normal brain: CD45^low^ that was also CD11b^+^, F11r^+^, Ly6G^neg^, Sell^neg^, CD3^neg^, CD19^neg^, and NK1.1^neg^. FACS samples were sorted directly into TRIzol (Life Technologies Corporation, Carlsbad, CA) for total RNA extraction.

### Gene expression analysis

Gene expression datasets were identified by specifically choosing only studies that performed gene expression analysis of both microglia and peripheral monocyte/macrophage populations at the same time, in order to minimize variation across sample preparation and analysis between laboratories. Datasets used for the meta-analysis included GSE46686 [33], GSE46690 [33], SRX424925 [22], GSE48579 [7], and GSE86573 [5] (Table 1).

**Table 1:** Gene expression datasets used for the meta-analysis

Cell Type	Dataset	Tissue	Species	Platform	Data Format	Reference
microglia	GSM1134004 GSM1134006 GSM1134009 GSM1134010 GSM1134012 GSM1134015 GSM1134055 GSM1134056 GSM1134057	brain	mouse	Aroma Aroma Aroma Expression console Expression console Expression console RNA-seq RNA-seq RNA-seq	CEL / microarray CEL / microarray CEL / microarray CEL / microarray CEL / microarray CEL / microarray	Pong et al. 2013 [33]
peripheral monocytes/ macrophages	GSM1134005 GSM1134007 GSM1134008 GSM1134011 GSM1134013 GSM1134014 GSM1134052 GSM1134053 GSM1134054	Bone marrow	mouse	Aroma Aroma Aroma Expression console Expression console Expression console RNA-seq RNA-seq RNA-seq	CEL / microarray CEL / microarray CEL / microarray CEL / microarray CEL / microarray CEL / microarray	Pong et al. 2013 [33]
microglia	SRX424861 SRX424857	brain	mouse	RNA-seq RNA-seq		Hickman et al. 2013 [22]
peripheral monocytes/ macrophages	SRX424925 SRX424919 SRX424904 SRX424890 SRX424880 SRX424879 SRX424878	peritoneum	mouse	RNA-seq RNA-seq RNA-seq RNA-seq RNA-seq RNA-seq RNA-seq		Hickman et al. 2013 [22]
microglia	GSM1181585 GSM1181587 GSM1181589	brain	mouse	Affymetrix Affymetrix Affymetrix	CEL / microarray CEL / microarray CEL / microarray	Butovsky et al. 2014 [7]
peripheral monocytes/ macrophages	GSM1181579 GSM1181581 GSM1181583	spleen	mouse	Affymetrix Affymetrix Affymetrix	CEL / microarray CEL / microarray CEL / microarray	Butovsky et al. 2014 [7]
microglia	GSM2590424 GSM2590425 GSM2590426	brain	mouse	RNA-seq RNA-seq RNA-seq		(Bowman et al. 2016 [5]
peripheral monocytes/ macrophages	GSM2590427 GSM2590428 GSM2590429 GSM2590430 GSM2590431	blood	mouse	RNA-seq RNA-seq RNA-seq RNA-seq RNA-seq		(Bowman et al. 2016 [5]

### Microarray analysis

Raw data files were downloaded, and analyzed using R package limma. The raw data was first normalized (RMA normalization), and the two groups were contrasted (lmFit and eBayes functions) in order to obtain the fold changes and adjusted *p*-values between microglia and monocyte/macrophage samples. The gene lists were further filtered for significant differential expression between monocytes/macrophages and microglia using a fold change (log2) cutoff of 2 and an adjusted p-value cutoff of 0.01. Each microarray dataset was individually analyzed.

### RNA sequencing analysis

Processed data files were downloaded and analyzed using different approaches depending on the dataset. For normalized expression (fpkm) datasets, the values were first log transformed, and the fold changes calculated using limma (lmFit and eBayes function). For read counts datasets, the fold changes were calculated by DESeq2 package using default values. The gene lists were further filtered for significant differentially expressed genes between monocytes/macrophages and microglia using a fold change (log2) cutoff of 2 and adjusted *p*-value cutoff of 0.01. Each RNA sequencing dataset was individually analyzed.

### Meta-analysis of mouse RNA sequencing and microarray data

In order to compare all datasets, gene IDs were converted to gene symbols. Genes with increased expression in microglia or monocytes/macrophages were compared using the R package GeneOverlap to identify overlaps and intersections. We only selected genes as potential markers for each cell type that intersected in all of the datasets. A heat map (heatmap.2 function) was then constructed using the fold-change values (data were scaled) for all of these markers, and adjusted according to hierarchical clustering.

### Analysis of mouse single-cell RNA sequencing (scRNA-Seq)

Single cell data from the Tabular Muris Consortium was retrieved and analyzed [42]. All cells were labeled with the tissue of origin (brain myeloid cells or marrow) and processed with Seurat software (v2.3). The two datasets were normalized, scaled (data were regressed based on ERCC spiked-in controls), and aligned together (CCA dimension alignment). We generated t-SNEs from the aligned CCA dimensions, and violin plots were created to depict the expression of each of the markers on individual cells from the two tissues.

### Analysis of mouse RNA sequencing datasets from glioma-associated microglia and glioma-associated monocytes/macrophages

RNA sequencing datasets from high-grade glioma-associated microglia and monocytes/macrophages isolated from experimental RCAS or GL261 tumors were extracted from GSE86573 or directly from the published manuscript (Table 1) [5]. Log2 fold changes in glioma-associated microglia relative to monocytes/macrophages were calculated for all microglia signature (SGMic) and monocyte/macrophage signature (SGMac) genes, including conventionally used markers (*Cx3cr1, Cd11b, Cd45, Ccr2*), and the data plotted accordingly. Additionally, log2 fold changes in glioma-associated microglia relative to healthy microglia were calculated for all microglia signature (SGMic) genes and the data plotted accordingly.

### Quantitative RT-PCR validation

Total RNA was extracted from FACS-sorted acutely isolated monocytes using ReliaPrep™ RNA Miniprep System (Promega Corporation; Madison, Wisconsin, USA), and first strand cDNA synthesis was performed using the PrimeScript™ RT reagent Kit (Takara, Kusatsu, Shiga, Japan) according to the manufacturer’s instructions. Quantitative real-time PCR reactions to amplify 1 ng of total cDNA for the selected genes (Table 2) were performed in a 7500 Fast Real-Time thermocycler (Applied Biosystems, Carlsbad, USA) using the SYBR Select Master Mix (Applied Biosystems | Thermo Fisher Scientific, Waltham, Massachusetts, USA). CT values were normalized using hypoxanthine guanine phosphoribosyltransferase (*Hprt*). To ensure the specificity of each PCR product, melting curves were analyzed. The delta/delta C_T_-method was employed for analysis of relative expression.

**Table 2:** Quantitative RT-PCR primers

Gene	Forward Primer	Reverse Primer
*C3*	TGCCCCTTACCCCTTCATTC	CTCCAGCCGTAGGACATTGG
*Emilin2*	GCAGCTTGTGGAACTGCATC	TCGGTTGCTTCTGAGGGTTC
*F10*	GGTGAGTGAACCTTGCCCC	TGGCACGTTCCCGGTTAATA
*F5*	CACCCGTGATACCTGCGAAT	TCAGTGCGTTTGGTGAAGGT
*Fcrls*	CTTGTGAGGCTGAAAACGCC	GCCATTCACCAAACGCACTT
*Gda*	GACAGCGGCAAAATAGTGTTTCT	AGGCCTGGCATGAAGAACTC
*Gpr34*	CCTGGTCTAGGGAGTTTTGGG	GAGCAAAGCCAGCTGTCAAC
*Hp*	CACTTGGTTCGCTATCGCTG	TCCATAGAGCCACCGATGAT
*Hprt*	GATTAGCGATGATGAACCAGGTT	CCTCCCATCTCCTTCATGACA
*Mki67*	TGGTCACCATCAAGCGGAG	AGGCAGCTGGATACGAATGT
*Olfml3*	GCCGACTAGCTGCCTTAGAG	CCTCCCTTTCAAGACGGTCC
*P2ry12*	GCACGGACACTTTCCCGTAT	GCCTTGAGTGTTTCTGTAGGGTA
*P2ry13*	CCTCATCGCTTTCGACAGGT	GAACATCAGGGACCAGACGG
*Sell*	TCATGGTCACCGCATTCTCG	CTTCACGGGAGGACTTGACG
*Siglec-H*	ATGTCAGCTGCCCTCATATCC	CCTGTACCACATCTGCCAGG
*Slc2a5*	ACAGCTGGCACTTTGAGGAG	TTGCCAGAGCAAGGACCAAT
*Tmem119*	CGGTCCTTCACCCAGAGC	TCGCAAGTAGCAGCAGAGAC

### Protein extraction and mass spectrometry analysis

For each of the four independent proteomic runs, primary monocyte populations from four different male C57BL/6J mice were pooled and pelleted in PBS. The samples were solubilized in Laemmli buffer (LB) and subjected to SDS-PAGE. The proteome was focused into one gel band and processed as previously published [26, 39], with the use of an automated HTS PAL system (CTC Analytics, Switzerland). Peptides were extracted, purified and stored on reversed-phase (C18) StageTips [35]. Following elution, the peptides were lyophilized and resuspended in 0.1% Formic Acid / 3% Acetonitrile, prior to separation in a nano EasyLC 1200 (Thermo Fisher Scientific) with a 0.1 × 200 mm MonoCap C18 HighResolution Ultra column (GL Sciences, Japan) at a flow rate of 300 nL/min and a gradient from 5 to 95% B (80% Acenotrile, 0,1% Formic Acid) in 360 min. The UHPLC was coupled online to an Orbitrap Q Exactive plus mass spectrometer (Thermo Fisher Scientific) for mass spectrometry analysis. The mass spectrometer was set to acquire full-scan MS spectra (300–1700 *m/z)* at a resolution of 17.500 after accumulation to an automated gain control (AGC) target value of 1 × 10^6^ and maximum injection time of 20 ms, and was operated in a data-dependent acquisition mode, selecting the 10 most abundant ions for MS/MS analysis, with dynamic exclusion enabled (20 s). Charge state screening was enabled, and unassigned charge states and single charged precursors excluded. Ions were isolated using a quadrupole mass filter with a *1.2 m/z* isolation window, with a maximum injection time of 60 ms. HCD fragmentation was performed at a normalized collision energy (NCE) of 26. The recorded spectra were searched against a mouse database from Uniprot (January 2017) using the MaxQuant software package (Version 1.5.2.8) [12] (with fixed modifications set to carbamylation of cysteines and variable modifications set to methionine oxidation). Peptide tolerance was 20 ppm and the minimum ratio for LFQ was set to 2. The false-discovery rate was set to 1% on protein and peptide level. Statistical analysis of the data set was performed using R-statistical software package (version 3.4.1), Prodigy (v0.8.2) and Perseus software (version 1.6.0.7).

For the data analysis, proteins that were only identified by site or were potential contaminants were excluded. Only those proteins discovered in at least three biological replicates were used for column-wise analysis using a two-sample t-test and a Benjamini-Hodgberg-based FDR < 0.05.

### mRNA library preparation and RNA sequencing

Total RNA from flow-sorted cells was isolated by TRIzol-chloroform extraction. RNA samples were resuspended in Ambion Nuclease-free water (Life Technologies), snap frozen, and stored at -80 °C. Prior to RNA sequencing, RNA was treated with TURBO DNA-free kit (Invitrogen | Thermo Fisher Scientific, Waltham, Massachusetts, USA) and assessed using the Agilent Eukaryotic Total RNA 6000 and Quant-iT™ RNA assay kit on a Qubit™ Fluorometer (Life Technologies). cDNA was synthesized using the Ovation® RNA-Seq method, and the Illumina paired-end LT indexing protocol used to construct an Illumina library from 500 ng cDNA [19, 30]. Libraries were sequenced on an Illumina HiSeq, and15-22Mbp per lane of 100 basepair paired-end reads generated. RNA-Seq paired-end reads were processed using the TopHat suite [44] with Cufflinks [36, 37]. A fold-change and significance (< 0.05 False Discovery Rate, FDR) for every gene was generated using cuffdiff [43].

### Data and software availability

The previously unpublished datasets from glioma-associated microglia and macrophages using the RCAS model are now available on the NCBI Gene Expression Omnibus (GEO Accession Series GSE65868).

## Results and Discussion

### Meta-analysis of gene expression datasets from microglia and peripheral monocyte/macrophage populations

To identify a reliable set of markers that distinguishes microglia from peripheral monocytes/macrophages, we leveraged a series of published RNA sequencing and microarray datasets from adult mouse brain microglia and peripheral monocyte/macrophage populations isolated from mouse bone marrow, blood, spleen and peritoneum. We only included studies that performed gene expression analyses of both populations, in order to minimize variations in the processing of the different samples between laboratories and the RNA analysis platforms [5, 7, 22, 33]. Isolation protocols for microglia varied among the studies; however, microglia were commonly isolated by fluorescence-activated cell sorting (FACS) using CD11b and CD45 expression. We incorporated datasets of monocyte/macrophage populations from different tissue origins, since there were few published studies that performed simultaneous sequencing of microglia and monocyte/macrophage populations. As such, the selected datasets included RNA sequencing and microarray data from brainstem microglia (CD11b^+^CD45^low^Ly6G^−^) and bone marrow-derived macrophages (CD11b^+^CD115^+^Ly6G^−^) isolated by fluorescence-activated cell sorting [33], RNA sequencing of microglia (CD11b^+^CD45^+^) and peritoneal macrophages (CD11b^+^CD45^+^; [22]), microarray data from microglia (CD11b^+^CD45^low^) and spleen monocytes (CD11b^+^Ly6C^+^; [7]), and RNA sequencing of microglia (CD11b^+^CD45^+^Ly6G^−^ Ly6C^−^) and blood monocytes (CD11b^+^CD45^+^Ly6G^−^ Ly6C^+^ [5]).

**Fig. 1:**
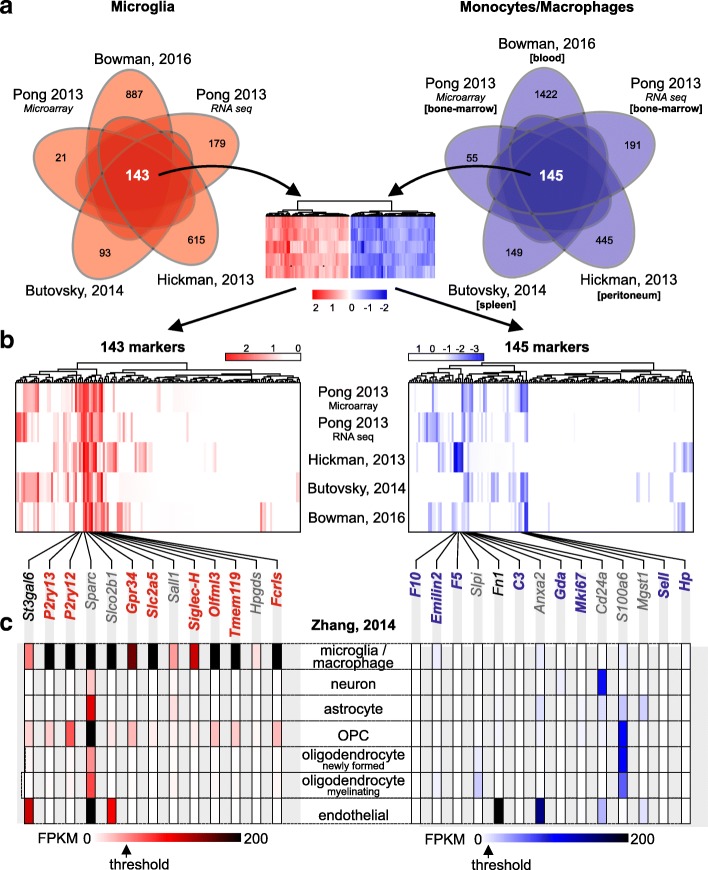
Meta-analysis of mouse gene expression datasets from microglia and peripheral monocyte/macrophage populations derived from bone marrow, blood, spleen and peritoneum. (**a**) Venn diagram representing commonly expressed genes across the analyzed RNA sequencing and microarray datasets for microglia (red) and peripheral monocytes/macrophages isolated from bone marrow, blood, spleen or peritoneum (blue). Bioinformatic analysis of the five different expression studies identified 143 microglia-specific genes and 145 genes specific for peripheral monocytes/macrophages. The number of microglia and peripheral monocyte/macrophage genes exclusively expressed in each of the datasets is represented. (**b**) Heat map representing gene expression (Z-score) of the identified 143 microglia-specific and 145 peripheral monocyte/macrophage-specific genes in each of the analyzed gene expression datasets. Hierarchical clustering identified modules of microglia-specific and of peripheral monocyte/macrophage-specific genes based on the dendrogram. For microglia, the module with the highest differential gene expression of identified microglia marker genes containing *St3gal6*, *P2ry13*, *P2ry12*, *Sparc*, *Slco2b1*, *Gpr34*, *Slc2a5*, *Sall1*, *Siglec-H*, *Olfml3*, *Tmem119*, *Hpgds* and *Fcrls* was selected for further analysis. For peripheral monocytes/macrophages, the two modules with highest differential gene expression containing the genes *F10*, *Emilin2*, *F5*, *Slpi*, *Fn1*, *C3*, *Anxa2*, *Gda*, *Mki67*, *Cd24a*, *S100a6*, *Mgst1*, *Sell* and *Hp* were selected for further analysis. (**c**) Representation of the expression levels for each of the selected microglia- and peripheral monocyte/macrophage-specific genes in different CNS cell types, including microglia/macrophages, neurons, astrocytes and oligodendrocyte precursor cells (OPCs), newly formed oligodendrocytes, myelinating oligodendrocytes and endothelial cells. FPKM values were extracted from the online database Brain-RNA-Seq (Zhang et al. 2014). The threshold FPKM expression value for excluding genes as microglia markers was set to 25, leading to elimination of *St3gal6*, *Sparc*, *Slco2b1*, *Sall1* and *Hpgds* as microglia markers for further validation. Threshold FPKM expression value for excluding genes as peripheral monocyte/macrophage markers was set to 10, leading to elimination of *Slpi*, *Fn1*, *Anxa2*, *Cd24a*, *S100a6* and *Mgst1* as monocyte/macrophage markers for further validation

Since the microarray and RNA sequencing data were analyzed using different methods and pipelines, we used the difference of gene expression (fold changes) between microglia and peripheral monocytes/macrophages within each dataset. Log-fold change values of enriched genes for each of the two populations were compared across the five different datasets (Fig. 1a). We identified 143 genes in microglia relative to peripheral monocytes/macrophages that were shared across the five analyzed studies. Next, hierarchical clustering was performed, revealing 13 microglia-enriched genes, including *St3gal6* (Type 2 lactosamine alpha-2,3-sialyltransferase), *P2ry13* (P2Y purinoceptor 13), *P2ry12* (P2Y purinoceptor 12), *Sparc* (Secreted Protein Acidic And Cysteine Rich), *Slco2b1* (Solute carrier organic anion transporter family member 2B1), *Gpr34* (Probable G-protein coupled receptor 34), *Slc2a5* (Solute carrier family 2, facilitated glucose transporter member 5), *Sall1* (Sal-like protein 1), *Siglec-H* (Sialic acid-binding Ig-like lectin H), *Olfml3* (Olfactomedin-like protein 3), *Tmem119* (Transmembrane protein 119), *Hpgds* (Hematopoietic prostaglandin D synthase), and *Fcrls* (Fc receptor-like S, scavenger receptor) (Fig. 1b, left panel). For the monocyte/macrophage populations derived from bone marrow, blood, spleen or peritoneum, 145 significantly enriched and specific genes shared across all five datasets were identified. Following hierarchical clustering, two clusters were selected, representing 14 genes with the highest expression differences relative to microglia, including *F10* (Coagulation factor X), *Emilin2* (Elastin Microfibril Interfacer 2), *F5* (Coagulation factor V), *Slpi* (Anti-leukoproteinase), *Fn1* (Fibronectin), *C3* (Complement C3), *Anxa2* (Annexin A2), *Gda* (Guanine deaminase), *Mki67* (proliferation marker protein Ki-67), *Cd24a* (CD24a antigen), *S100a6* (S100 Calcium Binding Protein A6), *Mgst1* (Microsomal glutathione S-transferase 1), *Sell* (L-selectin), and *Hp* (Haptoglobin) (Fig. 1b, right panel).

In order to evaluate the specificity of the identified markers for distinguishing microglia and monocytes/macrophages in the brain, we next assessed their expression in different CNS cell types using the Brain RNA-Seq transcriptome and splicing database [48]. Expression values were extracted for each gene within each marker set in microglia/macrophages, neurons, astrocytes, oligodendrocyte precursor cells, newly formed oligodendrocytes, myelinating oligodendrocytes and endothelial cells (Fig. 1c). For the set of microglia-enriched genes, we defined an expression threshold of 25 FPKM, such that any gene with a FPKM greater than 25 in any given CNS cell type other than microglia was excluded. As such, *St3gal6* and *Slco2b1* were eliminated from further analysis due to their high expression in endothelial cells, *Sparc* because of its high expression in all CNS cell types, and *Sall1* and *Hpgds* due to their low expression in microglia/macrophages. The 14 identified monocyte/macrophage markers exhibited low expression levels in microglia [48], consistent with the notion that there are few, if any, peripheral monocytes/macrophages in the healthy brain. We defined 10 FPKM reads as a threshold for the exclusion of monocyte/macrophage markers due to their expression in other brain cell types. For this reason, we eliminated *Slpi* due to its expression in newly formed and myelinating oligodendrocytes, *Fn1* and *Anxa2* due to their high expression in endothelial cells, and *Cd24a* for its high expression in neurons and endothelial cells. *S100a6* and *Mgst1* were also excluded, since they were highly expressed in astrocytes, OPCs and oligodendrocytes, astrocytes and endothelial cells, respectively. Taken together, a panel of eight specific microglia signature genes (SGmic: *P2ry13, P2ry12, Gpr34, Slc2a5, Siglec-H, Olfml3, Tmem119, Fcrls*) and eight specific peripheral monocyte/macrophage signature genes (SGmac: *F10, Emilin2, F5, C3, Gda, Mki67, Sell, Hp*) were identified.

Since these analyses included monocyte/macrophage populations derived from blood, bone marrow, spleen, and peritoneum, we also analyzed the expression of the identified SGmac genes across the different populations as shown in Additional file 1: Figure S1. While all markers were expressed in the different monocyte subsets, *Hp*, *Sell* and *Gda* were highly expressed in blood monocytes relative to spleen, bone marrow-derived or peritoneal macrophages. In addition, peritoneal macrophages exhibited high levels of *Fn1*, *Slpi*, *Emilin2* and *F10* expression, while *Hp*, *Sell*, *Mgst1* and *S100a6* were expressed at lower levels. Bone marrow-derived monocytes showed highest expression of *Cd24a* and *Mki67*, *C3* and *Fn1*.

### Validation of SGmic and SGmac in single-cell sequencing datasets

**Fig. 2:**
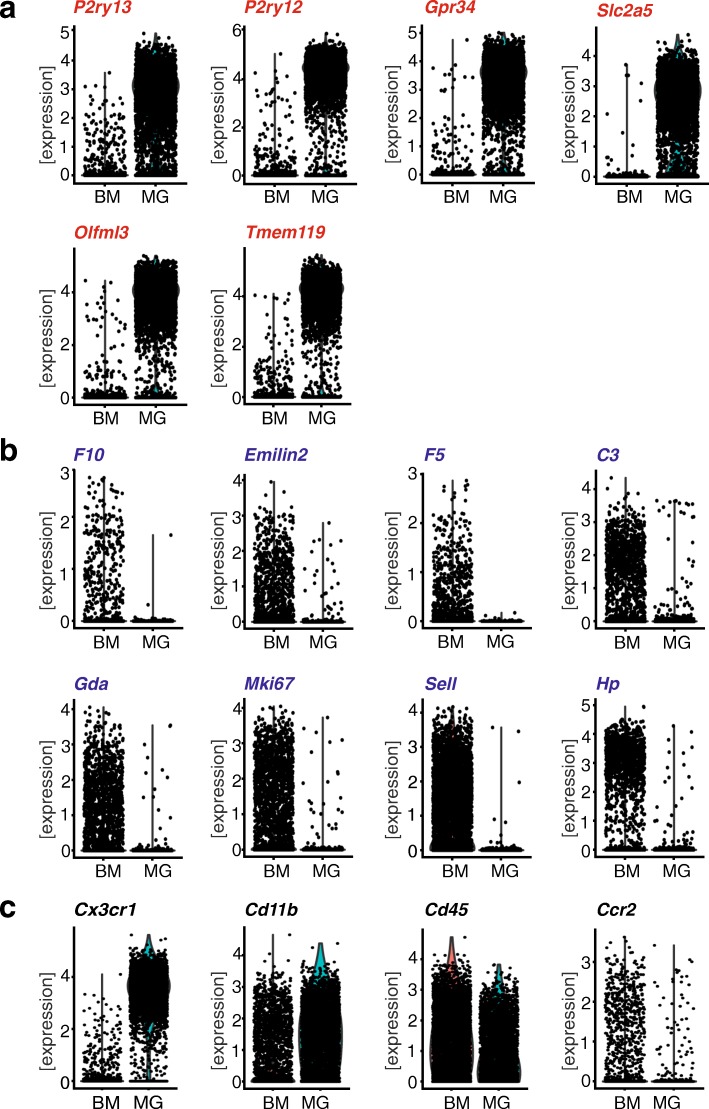
Validation of SGmic and SGmac gene expression in single-cell RNA sequencing datasets of brain myeloid cells (microglia) and bone marrow cells. Graph depicts normalized expression of single cell sequencing data of brain myeloid cells (termed MG for microglia) and bone marrow cells (BM) extracted from the Tabular Muris dataset [42] for (**a**) SGmic and (**b**) SGmac genes relative to the conventional markers *Cd11b*, *Cd45*, *Cx3cr1* and *Ccr2* (**c**)

To provide a second method for assessing the utility of these monocyte marker sets in discriminating microglia from peripheral monocytes/macrophages, we leveraged a recently published study using single-cell sequencing of microglia and bone marrow-derived cells [42]. For myeloid brain cells, data were collected from 4762 cells, while for bone marrow cells, data were derived from 5353 single cells. The bone marrow-derived cells were next sorted in silico for *Cd11b* and *Cd45* to identify monocytes; however, almost all of the cells expressed these two markers, making discrimination impossible. We next tried to sort for the fractalkine receptor (*Cx3cr1*), since peripheral monocytes/macrophages express only low levels of *Cx3cr1* [17, 24]. Unfortunately, *Cx3cr1* was expressed in myeloid brain cells, precluding its use to presort monocytes/macrophages from bone marrow-derived cells in silico. Thus, we compared the expression of the two marker sets in the brain myeloid fraction (termed microglia; MG) with the bone marrow cells (termed BM). Expression of each of the eight identified signature genes for microglia (Fig. 2a) and peripheral monocytes/macrophages (Fig. 2b) was normalized, and independently represented for each of the two populations.

Of the eight SGmic genes, six (*P2ry13*, *P2ry12*, *Gpr34*, *Slc2a5*, *Olfml3*, *Tmem119*) were present in the single-cell sequencing data, and all were enriched in the microglia population relative to the bone marrow-derived cells. *P2ry12*, *Olfml3* and *Tmem119* were enriched in nearly all of the sequenced cells, whereas *P2ry13*, *Slc2a5* and *Gpr34* were enriched, but not exclusively expressed in all of the sequenced microglia. *Siglec-H* and *Fcrls* were not present in the single cell RNA sequencing dataset. Conversely, all eight SGmac genes (*F10, Emilin2, F5, C3, Gda, Mki67, Sell, Hp*) were present in the bone marrow single cell sequencing dataset [42]: *Hp* and *C3* were enriched in the bone marrow cells compared to microglia; however, *C3* was also expressed in a small microglia population at elevated levels. All other monocyte/macrophage marker genes were enriched in bone marrow-derived cells relative to brain myeloid cells, with varying expression levels across the sequenced single cells. *Mki67* and *Gda* were enriched at higher levels than *Emilin2* and *F5*. *Sell* and *F10* expression was evenly distributed across the sequenced cells, with *F10* showing comparatively lower expression levels. In addition, the t-SNE distribution of microglia and bone marrow cells was examined, and the expression of the eight identified signature genes plotted for each of the populations as shown in Additional file 2: Figure S2. The brain myeloid cells clustered homogenously in the middle of the plot, and the expression of the eight SGmic genes correlated with that cluster. In contrast, the bone marrow cells formed six different clusters distributed at the periphery of the microglia cluster, of which, all of the SGmac genes were localized to at least two of these clusters.

We also examined the expression of four canonical microglia/macrophage markers (*Cd11b*, *Cd45*, *Cx3cr1* and *Ccr2*) within the Tabula Muris dataset (Fig. 2c). While *Cd11b* and *Cd45* were expressed in both myeloid brain cells and bone marrow-derived cells, *Cx3cr1* was enriched in microglia. Expression of the commonly used peripheral monocyte/macrophage marker *Ccr2* was only slightly enriched in the bone marrow cells, with very low levels of expression. Taken together, the classically used monocyte population markers underperformed as discriminatory genes relative to *Tmem119*, *P2ry12*, and *Olfml3* as microglia specific markers and *Hp*, *C3*, *Mki67*, *Gda* and *Sell* as monocyte/macrophage markers*.*

### SGmic and SGmac genes discriminate freshly isolated microglia from peripheral monocytes/macrophages

**Fig. 3:**
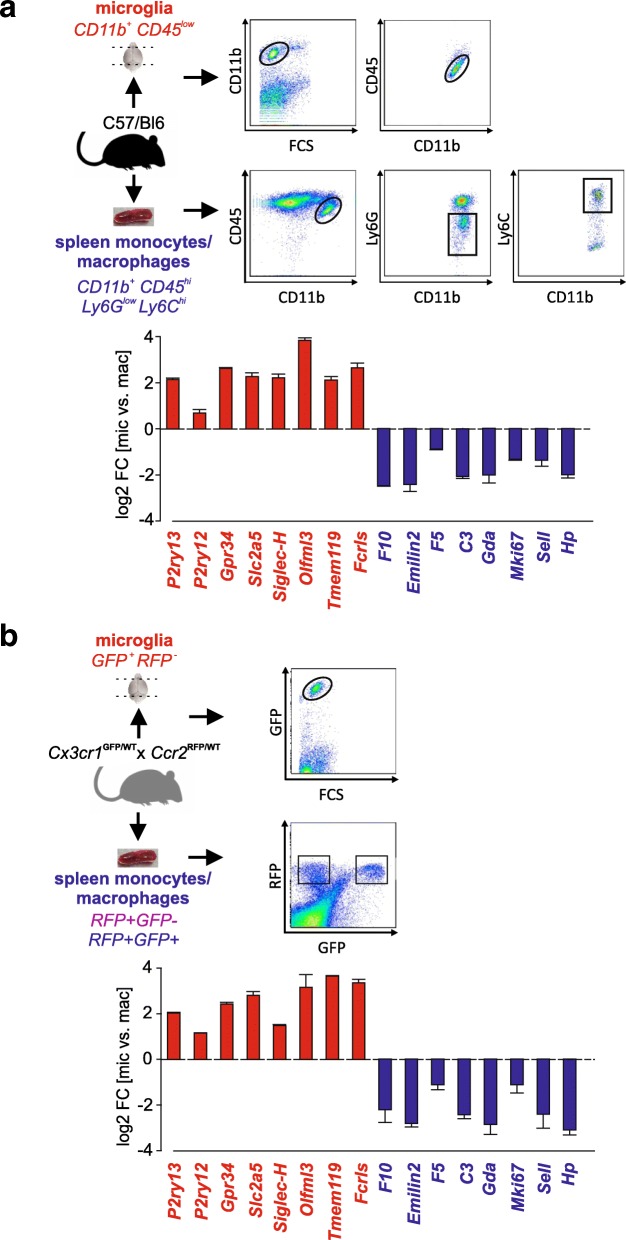
Validation of SGmic and SGmac genes by quantitative RT-PCR analysis in microglia and spleen monocytes/macrophages freshly isolated from two different mouse models. (**a**) Microglia and spleen monocytes/macrophages were freshly isolated from 12-weeks old male C57BL/6J WT mice by FACS. Microglia were first gated as CD11b^+^ cells against forward scatter (FSC) and subsequently selected as CD45^low^ expressing cells (microglia; red; CD11b + CD45^low^). Spleen monocytes/macrophages were first gated based on CD11b^+^ and CD45^high^ expression, followed by gating for Ly6G^low^ and Ly6C^high^ expression (spleen monocytes/macrophages; blue; CD11b + CD45^low^ Ly6G^low^ Ly6C^high^). Expression of SGmic (*P2ry13*, *P2ry12*, *Gpr34*, *Slc2a5*, *Siglec-H*, *Olfml3*, *Tmem119*, and *Fcrls*) and SGmac (*F10*, *Emilin2*, *F5*, *C3*, *Gda*, *Mki67*, *Sell*, *Hp*) genes was assessed in microglia (CD11b^+^ CD45^low^) and spleen monocytes/macrophages (CD11b^+^ CD45^high^ Ly6G^low^ Ly6C^high^) by quantitative RT-PCR. (**b**) Microglia and spleen monocytes were freshly isolated from 8 to 12 weeks old male *Cx3cr1*^GFP/WT^; *Ccr2*^RFP/WT^ mice by FACS. Microglia were gated as GFP-expressing cells against FSC (microglia; red; GFP^+^RFP^−^). Spleen monocytes/macrophages were isolated as RFP-expressing cells and sorted as two populations based on their GFP-expression levels as RFP^+^GFP^+^ (spleen monocytes/macrophages; blue) and RFP^+^GFP^−^ cells (spleen monocytes/macrophages; purple). Expression of SGmic (*P2ry13*, *P2ry12*, *Gpr34*, *Slc2a5*, *Siglec-H*, *Olfml3*, *Tmem119*, and *Fcrls*) and SGmac (*F10*, *Emilin2*, *F5*, *C3*, *Gda*, *Mki67*, *Sell*, *Hp*) genes was assessed in microglia (GFP^+^RFP^−^ cells) and spleen monocytes/macrophages (RFP^+^GFP^+^ cells) by quantitative RT-PCR. Bar graphs represent the log fold change expression of each gene normalized to *Hprt* and in the isolated microglia population relative to the peripheral monocytes/macrophage population (CD11b^+^ CD45^high^ Ly6G^low^ Ly6C^high^ or RFP^+^GFP^+^ cells; blue; *n* = 3)

To determine the discriminatory capabilities of these identified markers, we employed two different approaches. First, we isolated microglia as CD11b^+^CD45^low^ cells and spleen monocytes/macrophages as CD11b^+^CD45^high^Ly6G^low^Ly6C^high^ from 12-week-old male C57BL/6J mice by fluorescence-activated cell sorting (FACS), and determined their relative expression by RT-qPCR analysis (Fig. 3a). All eight SGmic markers were enriched and exclusively expressed in microglia relative to spleen monocytes/macrophages: *Olfml3, Fcrls*, and *Gpr34* exhibited the highest expression, with only *P2ry12* demonstrating very low expression levels in spleen monocytes/macrophages as shown in Additional file 3: Figure S3a. In addition, all eight SGmac markers were enriched in spleen monocytes/macrophages relative to microglia: *F10, Emilin2, C3, Gda* and *Hp* were exclusively and highly expressed in spleen monocytes/macrophages, whereas *F5, Mki67* and *Sell* were detected at low levels in microglia (Additional file 3: Figure S3a).

Using a second complementary method, we examined the SGmic and SGmac genes in *Cx3cr1*^GFP/WT^*;Ccr2*^RFP/WT^ mice, where green fluorescent protein (GFP) expression is driven by the fractalkine receptor (*Cx3cr1*) promoter, revealing microglia in the healthy brain as GFP^+^ cells. Conversely, red fluorescent protein (RFP) expression is controlled by the *Ccr2* promoter, allowing for the identification of peripheral monocytes/macrophages as RFP^+^ cells. While this mouse model was originally designed to distinguish CNS resident microglia from peripheral monocytes/macrophages, several studies have identified low *Cx3cr1* expression in the latter population [17, 24, 25]. Consistent with this observation, we detected a RFP^+^GFP^+^, as well as a RFP^+^GFP^−^, population in the *Cx3cr1*^GFP/WT^*;Ccr2*^RFP/WT^ spleen samples. Using this strain, we isolated microglia (GFP^+^RFP^−^ cells) from the healthy brain and two populations of spleen monocytes/macrophages (RFP^+^GFP^+^ and RFP^+^GFP^−^ cells) from 8 to 12-week-old male mice. We defined the RFP^+^GFP^+^ cells as the spleen monocyte/macrophage population (Fig. 3b).

Next, we determined the expression of the SGmic and SGmac marker sets across the three populations, focusing primarily on GFP^+^RFP^−^ microglia and RFP^+^GFP^+^ spleen monocytes/macrophages. All eight SGmic genes were enriched in the GFP^+^RFP^−^ microglia population relative to RFP^+^GFP^+^ cells, as well as to RFP^+^GFP^−^ cells (Fig. 3b; Additional file 3: Figure S3b). In these analyses, *Tmem119, Fcrls*, *Olfml3* and *Slc2a5* exhibited the highest levels of expression. As observed in C57BL/6J WT mice (Fig. 3a), low levels of *P2ry12* expression were detected in both spleen monocyte/macrophage populations (Additional file 3: Figure S3b). Conversely, the eight SGmac genes were enriched in the RFP^+^GFP^+^ population relative to GFP^+^RFP^−^ microglia, with *Emilin2, Gda and Hp* showing the highest expression levels. *Sell* was the only marker expressed at higher levels in the RFP^+^GFP^−^ population (Additional file 3: Figure S3b), but was still enriched in both isolated spleen monocyte/macrophage populations as compared to GFP^+^RFP^−^ microglia.

### Proteomic analysis confirms P2ry12, Tmem119, Slc2a5, and Fcrls as microglia markers, and Gda, Hp, C3, Mki67 and Emilin2 as monocyte/macrophage markers

**Fig. 4:**
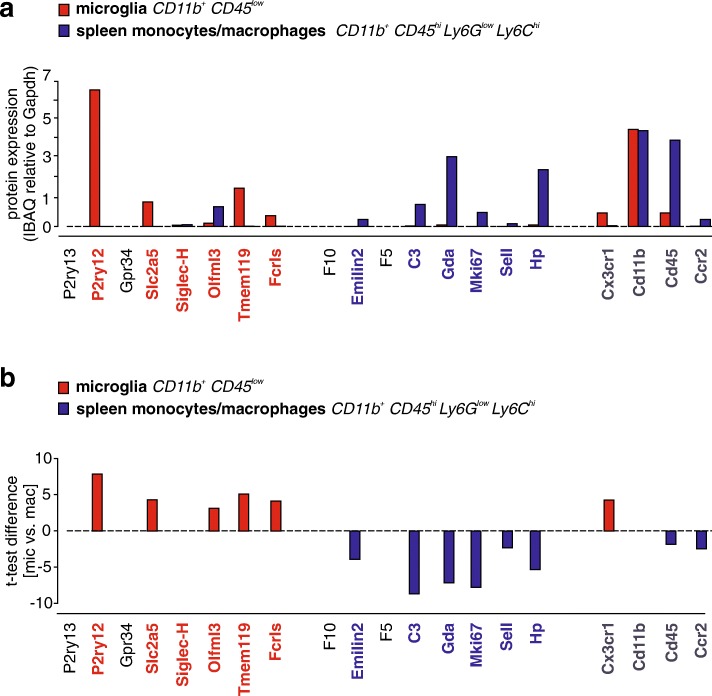
Protein expression of SGmic and SGmac markers in freshly isolated microglia and spleen monocytes/macrophages. (**a**) Protein expression of SGmic and SGmac genes and reference markers (Cx3cr1, Cd11b, CD45, Ccr2) in microglia and spleen monocytes/macrophages. IBAQ intensities of each protein normalized to Gapdh intensity are shown. (**b**) Proteomic data were analyzed by a column-wise analysis using a two-sample t-test and a Benjamini-Hodgberg-based FDR < 0.05. T-test difference of SGmic, SGmac and reference marker expression in microglia relative to spleen monocytes/macrophages is shown (*n* = 4)

To provide a third line of evidence for the discriminatory ability of the identified SGmic and SGmac gene sets, we sought to confirm their differential expression at the protein level. Proteomics data were generated from microglia (CD11b^+^CD45^low^) and spleen monocytes/macrophages (CD11b^+^CD45^high^Ly6G^low^Ly6C^high^) isolated by FACS from 12-week-old naïve C57BL/6J male mice. Protein expression levels of the SGmic and SGmac genes were calculated from the measured IBAQ intensities and normalized to Gapdh (Fig. 4a), and the t-test differences in protein expression determined (Fig. 4b). Since P2ry13 and Gpr34 could not be identified in the proteomic analysis, they were excluded. Enrichment of P2ry12, Slc2a5, Olfml3, Tmem119, and Fcrls protein levels were observed in microglia relative to spleen monocytes/macrophages. When normalized to Gapdh, P2ry12 expression was highest in microglia, followed by Tmem119, Slc2a5 and Fcrls. Olfml3 protein expression, however, was higher in spleen monocytes/macrophages than in microglia. The switch in quantification can be explained by the method of protein extraction for proteomic analysis. Since Olfml3 is a secreted protein, only the intracellular amount of the protein can be accurately recovered and quantified. When compared to spleen monocytes/macrophages, P2ry12 showed the highest t-test difference, followed by Tmem119, Slc2a5, Fcrls and Olfml3.

The monocyte/macrophage markers F5 and F10 could not be detected in the proteomics analysis, which might be explained by the fact that both are secreted proteins and intracellular protein levels might fall below proteomic detection levels. Normalized to Gapdh, Gda and Hp showed the highest protein expression levels in peripheral monocytes/macrophages, followed by C3, Mki67 and Sell. For Gda and Hp, very low protein expression in microglia was observed. Relative to microglia, C3, Mki67, Gda and Hp protein levels showed the highest t-test difference, followed by Emilin2 and Sell. As a reference, protein expression of the conventionally used microglia/macrophage markers, Cx3cr1, Cd11b, Cd45 and Ccr2, were also analyzed. Cd11b protein was highly expressed in both microglia and spleen monocytes/macrophages, Cx3cr1 was enriched in microglia, and Cd45 and Ccr2 were mildly enriched in peripheral spleen monocytes/ macrophages when compared to microglia. Taken together, we provide the first transcriptomic and proteomic evidence for Hp, Gda, Sell, C3, Mki67 and Emilin2 as specific markers for peripheral monocytes/macrophages and P2ry12, Tmem119, Slc2a5 and Fcrls as microglia-specific markers.

### SGmic and SGmac genes discriminate between glioma-associated microglia and monocytes/macrophages

Using the SGmic and SGmac gene sets, we next explored their utility for discriminating between microglia and infiltrated monocytes/macrophages in the setting of brain cancer. For these studies, we employed datasets derived from two different experimental murine glioblastoma models, the induced RCAS-TVA system [20] and the GL261 glioma explant system [38].

**Fig. 5:**
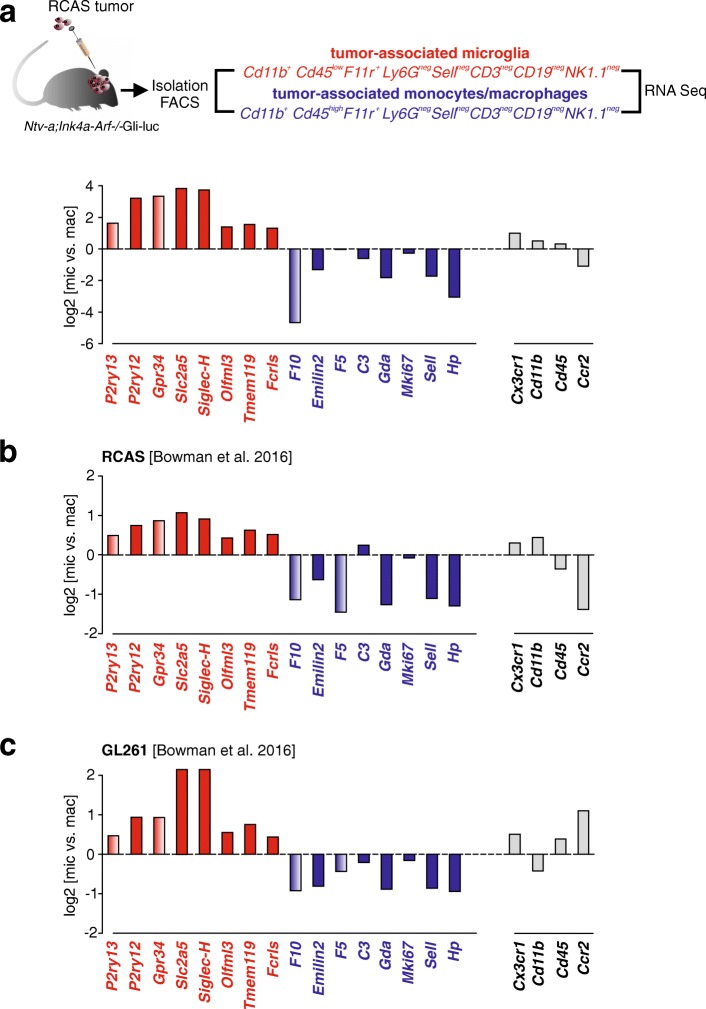
Expression of SGmic and SGmac genes in glioma-associated microglia and monocytes/macrophages isolated from two different experimental mouse glioma models. (**a**) RCAS tumors were generated by injection of RCAS-PDFGB into *Ntv-a*;*Ink4a-Arf−/−*;Gli-luc mice, and after 5 weeks, glioma-associated microglia were isolated as CD11b^+^, CD45^low,^ F11r^+^, Ly6G^neg^, Sell^neg^, CD3^neg^, CD19^neg^, NK1.1^neg^ cells, while glioma-associated monocytes/macrophages were isolated as CD11b^+^, CD45^high^, F11r^+^, Ly6G^neg^, Sell^neg^, CD3^neg^, CD19^neg^, NK1.1^neg^ cells by FACS. Graph shows RNA sequencing results of the two populations as log_2_-fold change expression of glioma-associated microglia to monocytes/macrophages for SGmic (*P2ry13*, *P2ry12*, *Gpr34*, *Slc2a5*, *Siglec-H*, *Olfml3*, *Tmem119*, *Fcrls*) and SGmac (*F10*, *Emilin*, *F5*, *C3*, *Gda*, *Mki67*, *Sell* and *Hp*) genes, as well as the reference genes (*Cx3cr1, CD11b, CD45, Ccr2*). Genes not detected in the previous proteomic analysis (see Fig. 4; SGmic: *P2ry13, Gpr34* and SGmac: *F10, F5*) are colored with gradients. Graphs show log_2_-fold change expression of the SGmic and SGmac genes in glioma-associated microglia versus monocytes/macrophages isolated from (**b**) RCAS and (**c**) GL261 tumors derived from published sequencing data [5]. Glioma-associated microglia were isolated from RCAS tumors based on CD45^+^CD11b^+^Ly6G^−^Ly6C^−^ TdTomato^+^ GFP^−^ expression, glioma-associated monocytes/macrophages were isolated as CD45^+^CD11b^+^Ly6G^−^Ly6C^−^ TdTomato^−^ GFP^+^ cells. GL261 glioma-associated microglia were isolated as CD45^+^CD11b^+^Ly6G^−^Ly6C^−^TdTomato^+^ cells and glioma-associated monocytes/macrophages were isolated as CD45^+^CD11b^+^Ly6G^−^Ly6C^−^ TdTomato^−^ cells

First, we generated RCAS/TVA-induced tumors in *Ntv-a;Ink4a-Arf*^−/−^;Gli-luc mice by RCAS-mediated expression of PDGFB, and subsequently isolated tumor-associated microglia and monocytes/macrophages based on CD11b^+^, CD45^low,^ F11r^+^, Ly6G^neg^, Sell^neg^, CD3^neg^, CD19^neg^, and NK1.1^neg^ (microglia) and CD11b^+^, CD45^high^, F11r^+^, Ly6G^neg^, Sell^neg^, CD3^neg^, CD19^neg^, and NK1.1^neg^ (monocytes/macrophages) gating. RNA sequencing was performed, and the log2 fold changes in expression were calculated for each gene (Fig. 5a). In the RCAS/TVA system, all SGmic genes (*P2ry13, P2ry12, Gpr34, Slc2a5, Siglec-H, Olfml3, Tmem119, Fcrls*) were enriched in glioma-associated microglia relative to glioma-associated monocytes/macrophages, with *Slc2a5*, *Siglec-H*, *Gpr34* and *P2ry12* showing the highest differential expression. Similarly, the SGmac markers *F10* and *Hp* were increased in glioma-associated monocytes/macrophages (log2-fold changes = 3–4.7), whereas *Emilin2*, *Gda* and *Sell* were slightly increased (log2-fold changes = 1.1–1.8) and *C3* and *Mki67* only barely enriched. *F5* did not show any changes in gene expression between glioma-associated microglia and monocytes/macrophages. The canonical reference genes, *Cx3cr1*, *Cd11b* and *Cd45* were all enriched in microglia, while the classical monocyte/macrophage marker *Ccr2* was enriched in monocytes/macrophages isolated from RCAS tumors.

As further confirmation, we leveraged an independently-generated RNA sequencing dataset generated by the Joyce laboratory using the identical RCAS platform [5]. In this study, glioma-associated microglia were isolated from RCAS-induced gliomas based on CD45^+^CD11b^+^Ly6G^−^Ly6C^−^TdTomato^+^GFP^−^ expression, whereas glioma-associated monocytes/macrophages were isolated as CD45^+^CD11b^+^Ly6G^−^Ly6C^−^TdTomato^−^GFP^+^ cells. Data were extracted, and the log2-fold change expression of glioma-associated microglia calculated relative to glioma-associated monocytes/macrophages (Fig. 5b). All eight SGmic genes were enriched in glioma-associated microglia in this RCAS dataset, with *Slc2a5*, *Siglec-H*, *Gpr34* and *P2ry12* exhibiting the highest expression, followed by *Tmem119*, *Fcrls*, *P2ry13* and *Olfml3*, similar to the experimental data presented in Fig. 5a. The peripheral monocyte/macrophage marker genes *F5*, *Gda, Hp*, *Sell* and *F10* were strongly enriched in glioma-associated monocytes/macrophages, followed by *Emilin2*. *Mki67* expression did not show a significant difference between glioma-associated microglia and monocytes/macrophages, while *C3* was slightly enriched in the glioma-associated microglia fraction. Similarly, *Cx3cr1* and *Cd11b* were only slightly enriched in glioma-associated microglia, while *Cd45* and *Ccr2* were enriched in glioma-associated monocytes/macrophages.

We then employed a second published RNA sequencing dataset derived from the GL261 glioma model system that was also analyzed by the Joyce laboratory [5]. GL261 glioblastoma tumors were implanted in wild-type C57BL/6J mice and glioma-associated microglia were isolated as CD45^+^CD11b^+^Ly6G^−^Ly6C^−^TdTomato^+^ cells by FACS sorting, while glioma-associated monocytes/macrophages were isolated as CD45^+^CD11b^+^Ly6G^−^Ly6C^−^ TdTomato^−^ cells. As above, we calculated log2-fold changes in gene expression (Fig. 5c), and found that all eight identified microglia and peripheral monocyte/macrophage markers were significantly enriched in the glioma-associated microglia and monocyte/macrophage populations, respectively. Similar to the RCAS-TVA tumors, *Slc2a5* and *Siglec-H* were expressed at the highest levels in GL261 glioma-associated microglia, followed by *P2ry12* and *Gpr34*, while *F10*, *Emilin2*, *Gda*, *Sell* and *Hp* showed the highest expression in glioma-associated monocytes/macrophages. As previously seen, *C3* and *Mki67* were only barely enriched in glioma-associated monocytes/macrophages. While *Cd11b* was enriched in GL261-derived monocytes/macrophages, the other classical monocyte marker genes (*Cx3cr1*, *Cd45* and *Ccr2*) showed enrichment in GL261-derived microglia.

In addition, we assessed whether and how the pathologic condition of glioma affects SGmic gene expression in microglia by calculating the log2 fold changes of SGmic gene expression between glioma-associated microglia and healthy microglia in both glioma models and for all three RNA sequencing datasets as shown in Additional file 4: Figure S4. All SGmic genes showed a decrease in their expression levels in glioma-associated microglia as compared to healthy microglia across glioma models and datasets (log2-fold changes = 0.05-5.0), except for *Fcrls* in the RCAS dataset published by Bowman et al. [5]. Here, *Slc2a5* and *P2ry12* showed the strongest decrease across datasets, while *Olfml3* and *Tmem119* expression was least affected (*Slc2a5* = − 0.68 to − 5.0; *P2ry12* = − 0.61 to − 1.89; *Olfml3* = − 0.05 to − 1.17; *Tmem119* = − 0.50 to − 1.99).

### The value of the SGmic and SGmac markers for understanding CNS pathogenesis

Myeloid cells are highly dynamic cells whose transcriptomes are highly influenced by specific disease states, limiting their utility as reliable and stable cell identity markers. As such, microglia harbor gene expression patterns that reflect specific neuropathological conditions [21, 23]. For example, in experimental mouse models of Alzheimer’s disease (AD) and amyotrophic lateral sclerosis, unique microglia gene expression patterns have been reported [11, 27, 29]. Moreover, even within the same disease state (e.g., AD), microglia change their transcriptomes during the evolution of the pathologic process, reflecting the trajectory of cellular reprogramming in response to neurodegeneration and other CNS pathologies [31]. These temporal and spatial changes in microglial gene expression, and likely function, in the setting of CNS disease support the need for discriminatory markers that distinguish resident microglia from infiltrating monocyte/macrophage populations, so that the relative contributions of each monocyte population can be studied in greater detail.

The commonly used markers for distinguishing microglia from infiltrated monocytes/macrophages in the mouse system, including CD45, CX3CR1, and CCR2, have limitations that reflect their relative expression levels, which are presumed not to vary as a function of cellular context. In this regard, CD45 expression is frequently employed to distinguish microglia from peripheral monocytes/macrophages in FACS-based monocyte cell separations. However, this distinction relies upon gating the cells for differential expression levels, where microglia express low to intermediate levels and blood-derived monocytes/macrophages express high levels [15]. Obtaining clean separations is therefore dependent on the overlap between the different CD45-expressing populations, and does not consider that these levels could vary under pathological conditions. Germane to this latter issue, glioma-associated microglia increase CD45 expression in vivo, rendering them indistinguishable from CD45^high^-expressing monocytes/macrophages [32]. In addition, we found that *Cd45* expression was enriched in glioma-associated microglia relative to glioma-associated monocytes/macrophages (Fig. 5a, c). Similarly, while Cx3cr1 is often considered to be microglia-specific, circulating monocytes and resident tissue macrophages can also express Cx3cr1*.* Additionally, Ccr2, a blood-derived macrophage marker [16–18, 25], can be induced in microglia following lipopolysaccharide (LPS) treatment or reduced in blood-derived monocytes/macrophages once they enter the brain in the context of CNS pathology [1, 4, 11, 40, 47]. This problem is further underscored by the observation that *Ccr2* was enriched in glioma-associated monocytes/macrophages in both RCAS-tumor datasets, while it was enriched in glioma-associated microglia isolated from the GL261-tumors (Fig. 5). Finally, we have previously shown that peripheral monocytes/macrophages acquire expression of a microglia-specific gene (F11r) upon entry into the brain using an experimental model of graft versus host disease and, rendering infiltrating monocytes/macrophages indistinguishable from resident microglia [33].

Similarly, several studies postulated novel and exclusive markers for identifying microglia in disorders affecting the CNS, including *Tmem119* [3] and *P2ry12* [7]. As such, *TGF-ßR1*, *Fcrls*, *Gpr34*, *Sall1* and *P2ry12* [7], as well as *Siglec-H* [28], have been reported to be expressed at higher levels in microglia than in peripheral monocytes/macrophages. *CD49D/Itga4* has also been described as a specific marker for bone-marrow derived macrophages due to its transcriptional suppression in microglia, and has been shown to separate the two cell populations in murine and human tumors [5]. In addition, *TREM2* has similarly been suggested to distinguish infiltrated monocytes/macrophages from microglia [14]. However, none of these markers has been accepted as a universal standard.

The lack of a common set of markers to distinguish microglia from peripheral monocytes/macrophages that infiltrate the CNS has limited our understanding of the relative contributions of each of these monocyte populations to neurologic disease pathogenesis. In the present study, we employed an unbiased and comprehensive meta-analytic approach, combined with numerous experimental validations to identify two sets of highly reliable markers for microglia (SGmic) and peripheral monocytes/macrophages (SGmac). These SGmic and SGmac gene sets were then leveraged to separate microglia from infiltrating monocytes/macrophages in two different experimental mouse models of high-grade glioma. Within these marker sets, *P2ry12*, *Tmem119*, *Slc2a5* and *Fcrls* performed best to discriminate microglia from other cell types, while *Emilin2*, *Gda, Hp* and *Sell* were the best markers for peripheral monocytes/macrophages. Throughout all investigated conditions and approaches, these markers were more reliable and performed better than the commonly used microglia/macrophage discriminators, underscoring their utility for discriminating these myeloid cell populations in both health and glioma and arguing for their use in future studies. Despite the observation that SGmic gene expression changed in glioma-associated microglia, and that LPS exposure decreased the expression of *P2ry12*, *Tmem119*, *Fcrls* and *Olfml3* [3], the SGmic genes still outperformed the commonly used discriminators.

Based on the ability of our prime candidate microglia signature genes (*P2ry12*, *Slc2a5*, *Tmem119 and Fcrls*), as well as our top candidate marker genes for peripheral monocytes/macrophages (*Gda* and *Hp*, *Sell* and *Emilin2*), to stably distinguish these two populations in the normal brain, and in the context of high-grade glioma, it is interesting to note that a preliminary analysis indicates that *P2ry12*, *Slc2a5* and *Tmem119* genes are expressed in glioma-associated microglia isolated from a murine low-grade glioma model [41]. Thus, besides further proving the validity of SGmic and SGmac genes as reliable markers used in the field of glioma research, their applicability might also be explored in the broader context of other CNS diseases.

While *Tmem119* and *P2ry12* have already been shown to reliably identify human healthy microglia [3, 7], our results suggest that the other SGmic genes (*P2ry13*, *Gpr34*, *Slc2a5*, *Siglec*-*H*, *Olfml3*, *Fcrls*) may also serve as human microglia markers. Moreover, future studies might explore whether *Tmem119*, *P2ry12* (and potentially other SGmic genes) might possess the ability to distinguish glioma-associated microglia from glioma-associated monocytes/macrophages in human glioma tissue.

Since the SGmic genes (*P2ry12*, *Slc2a5, Tmem119* and *Fcrls*) and SGmac genes (*Gda* and *Hp*, *Sell* and *Emilin2*) were validated at the protein level and are predicted to be expressed at the plasma membrane, it becomes possible to consider them for future protein-based applications, such as Western blotting, immunocytochemistry, FACS analysis, and potentially for generating new mouse reporter or Cre driver lines.

## Conclusions

Using large meta-analytic approach, we identified a robust panel of microglia and peripheral monocyte/macrophage markers, which were independently validated at the RNA and protein levels. The value of these discriminating marker sets was further explored in the setting of glioma, where they distinguished glioma-associated microglia from macrophages in two mouse glioblastoma models. Future studies employing these discriminatory genes/proteins to separate monocyte populations may facilitate the discovery of novel and distinct functions for microglia and infiltrating monocytes/macrophages in CNS disease.

## Additional files

Additional file 1: **Figure S1.**. Expression levels of selected differentially expressed macrophage marker genes after hierarchical clustering in peripheral monocyte/macrophage subpopulations isolated from blood, spleen, peritoneum and bone marrow. (a) The differentially-expressed SGmac genes, which were identified in cluster 1 (*Cd24, Mki67, Gda, Anxa2, C3, Fn1, Slpi, Emilin2, F10*) following hierarchical clustering of the 145 significantly enriched and specific peripheral monocyte/macrophage genes shared across all five datasets, are shown. Expression is shown as the log2 fold change of expression of the peripheral monocyte/macrophage subpopulations isolated from blood (dark green; [5]), spleen (light green; [7]), peritoneum (light blue; [22]) and bone marrow (dark grey; [33]) compared to microglia for each of the datasets. For bone marrow-derived monocyte/macrophages, the RNA-sequencing dataset from Pong et al. is shown [33]. (b) The differentially-expressed SGmac genes, which were identified in cluster 2 (*Hp, Sell, Mgst1 and S100a6*) following hierarchical clustering of the 145 significantly enriched and specific peripheral monocyte/macrophage genes shared across all five datasets, are shown. Expression is shown as the log2 fold change of expression of the peripheral monocyte/macrophage subpopulations isolated from blood (dark green; [5]), spleen (light green; [7]), peritoneum (light blue; [22]) and bone marrow (dark grey; [33]) compared to microglia for each of the datasets. For bone marrow- derived monocyte/macrophages, the RNA-sequencing dataset from Pong et al. is shown [33]. (PDF 393 kb)

Additional file 2: **Figure S2.** Spatial visualization, clustering and expression of SGmic, SGmac and classical monocyte/macrophage marker genes in single cell sequencing data derived from brain myeloid and bone marrow cells. (a) t-distributed Stochastic Neighbor Embedding (*t-SNE*) spatial visualization and clustering of brain myeloid single cells (microglia; turquoise) dataset and bone marrow single cells (red) dataset derived from the single cell sequencing data of the Tabula Muris Consortium [42]. The right panel depicts clusters 1–16 represent all different cell populations detected by automatic clustering (Seurat FindCluster function). (b) t-SNEs showing the expression of the SGmic genes within the spatial distribution of brain myeloid and bone marrow cells. Identified SGmic genes from the analyzed single cell sequencing dataset comprise *P2ry13*, *P2ry12*, *Gpr34*, *Slc2a5*, *Olfml3*, *Tmem119.* (c) t-SNEs showing the expression of the SGmac genes (*F10*, *Emilin2*, *F5*, *C3*, *Gda*, *Mki67*, *Sell*, *Hp*) within the spatial distribution of brain myeloid and bone marrow cells. (d) t-SNEs showing the expression of the canonical monocyte/macrophage markers (*Cx3Cr1*, *Cd11b*, *Cd45* and *Ccr2*) within the spatial distribution of brain myeloid and bone marrow cells. (PDF 14645 kb)

Additional file 3: **Figure S3.** Validation of SGmic and SGmac genes in microglia and peripheral monocytes/macrophages freshly isolated from two different mouse models. (a) Microglia (CD11b^+^ CD45^low^; red) and circulating spleen monocytes (CD11b^+^ CD45^high^ Ly6G^low^ Ly6C^high^; blue) were freshly isolated from 12-week-old male C57/Bl6 WT mice by FACS and the expression of SGmic genes (*P2ry13*, *P2ry12*, *Gpr34*, *Slc2a5*, *Siglech*, *Olfml3*, *Tmem119*, and *Fcrls; red*) and SGmac genes (*F10*, *Emilin2*, *F5*, *C3*, *Gda*, *Mki67*, *Sell*, *Hp;* blue) determined by quantitative RT-PCR. Bar graphs represent the fold change expression of marker genes normalized to *Hprt*, where SGmic genes (red) are shown in relation to the spleen monocyte/macrophage population (CD11b^+^ CD45^high^ Ly6G^low^ Ly6C^high^; blue) and SGmac genes (blue) compared to microglia (*n* = 3). For statistical analysis, unpaired t-tests were performed. * = *P* < 0.05; ** = *P* < 0.01; *** = *P* < 0.001. (b) Microglia and spleen monocytes/macrophages were freshly isolated from 8 to 12-week-old male *Cx3cr1*^GFP/WT^;*Ccr2*^RFP/WT^ mice by FACS, GFP^+^RFP^−^ cells representing microglia (red), RFP^+^GFP^+^ (blue) and RFP^+^GFP^−^ (purple) cells representing spleen monocytes/macrophages. The expression of SGmic genes (*P2ry13*, *P2ry12*, *Gpr34*, *Slc2a5*, *Siglech*, *Olfml3*, *Tmem119*, and *Fcrls;* red) and SGmac genes (*F10*, *Emilin2*, *F5*, *C3*, *Gda*, *Mki67*, *Sell*, *Hp;* blue) was determined by quantitative RT-PCR. Bar graphs represent the fold change expression of each gene normalized to *Hprt*, where SGmic genes (red) are shown in relation to spleen monocytes/macrophages (RFP^+^GFP^+^; blue) and SGmac genes compared to microglia (GFP^+^RFP^−^ cells; *n* = 3). For statistical analysis, one-way ANOVA following Bonferroni’s multiple comparison test was performed. * = *P* < 0.05; ** = *P* < 0.01; *** = *P* < 0.001. (PDF 401 kb)

Additional file 4: **Figure S4.** Expression of SGmic genes in glioma-associated microglia as compared to healthy microglia in RNA sequencing datasets derived from healthy, RCAS glioma or GL261 glioma mice. (a) The log-fold change expression of SGmic genes (*P2ry13*, *P2ry12*, *Gpr34*, *Slc2a5*, *Siglec*-H, *Olfml3*, *Tmem119*, *Fcrls*) in glioma-associated microglia isolated from experimental RCAS tumors compared to microglia isolated from healthy control brains is shown. Expression data were extracted from RNA sequencing data generated by our group. (b) The log-fold change expression of SGmic genes (*P2ry13*, *P2ry12*, *Gpr34*, *Slc2a5*, *Siglec*-H, *Olfml3*, *Tmem119*, *Fcrls*) in glioma-associated microglia isolated from RCAS tumors compared to microglia isolated from healthy control brains is shown. Expression data were extracted from published RNA sequencing data [5]. (c) Graph shows the log-fold change expression of SGmic genes (*P2ry13*, *P2ry12*, *Gpr34*, *Slc2a5*, *Siglec*-H, *Olfml3*, *Tmem119*, *Fcrls*) in glioma-associated microglia isolated from GL261 tumors as compared to microglia isolated from healthy control brains. Expression data were extracted from published RNA sequencing data [5]. (PDF 394 kb)
