# Correction to: Comprehensive gene expression meta-analysis identifies signature genes that distinguish microglia from peripheral monocytes/macrophages in health and glioma

**DOI:** 10.1186/s40478-019-0875-3

**Published:** 2020-01-08

**Authors:** Verena Haage, Marcus Semtner, Ramon Oliveira Vidal, Daniel Perez Hernandez, Winnie W. Pong, Zhihong Chen, Dolores Hambardzumyan, Vincent Magrini, Amy Ly, Jason Walker, Elaine Mardis, Philipp Mertins, Sascha Sauer, Helmut Kettenmann, David H. Gutmann

**Affiliations:** 1 Max Delbrück Center for Molecular Medicine in the Helmholtz Association, Berlin, Germany; 2 Department of Neurology, Washington University School of Medicine, Box 8111, 660 S. Euclid Avenue, St. Louis, MO 63110 USA; 3 Department of Pediatrics, Emory University, Atlanta, GA USA; 4 McDonnell Genome Institute, Washington University School of Medicine, St. Louis, MO USA

**Correction to: Acta neuropathol commun (2019) 7: 20**

**https://doi.org/10.1186/s40478-019-0665-y**

The original publication of this article [1] contained 3 minor errors in Figs. 1, 3 and 5. In this correction article the updated figures are published. The figure captions describe the updated information in these figures.

**Fig. 1:**
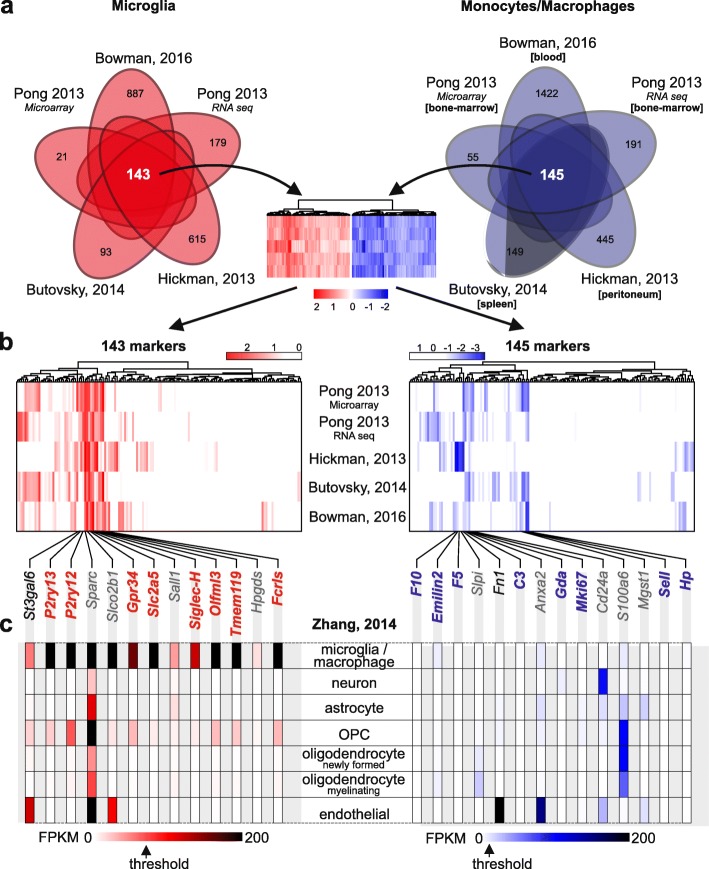
The threshold FPKM expression value for excluding genes as microglia markers is 70 (panel **c**)

**Fig. 3:**
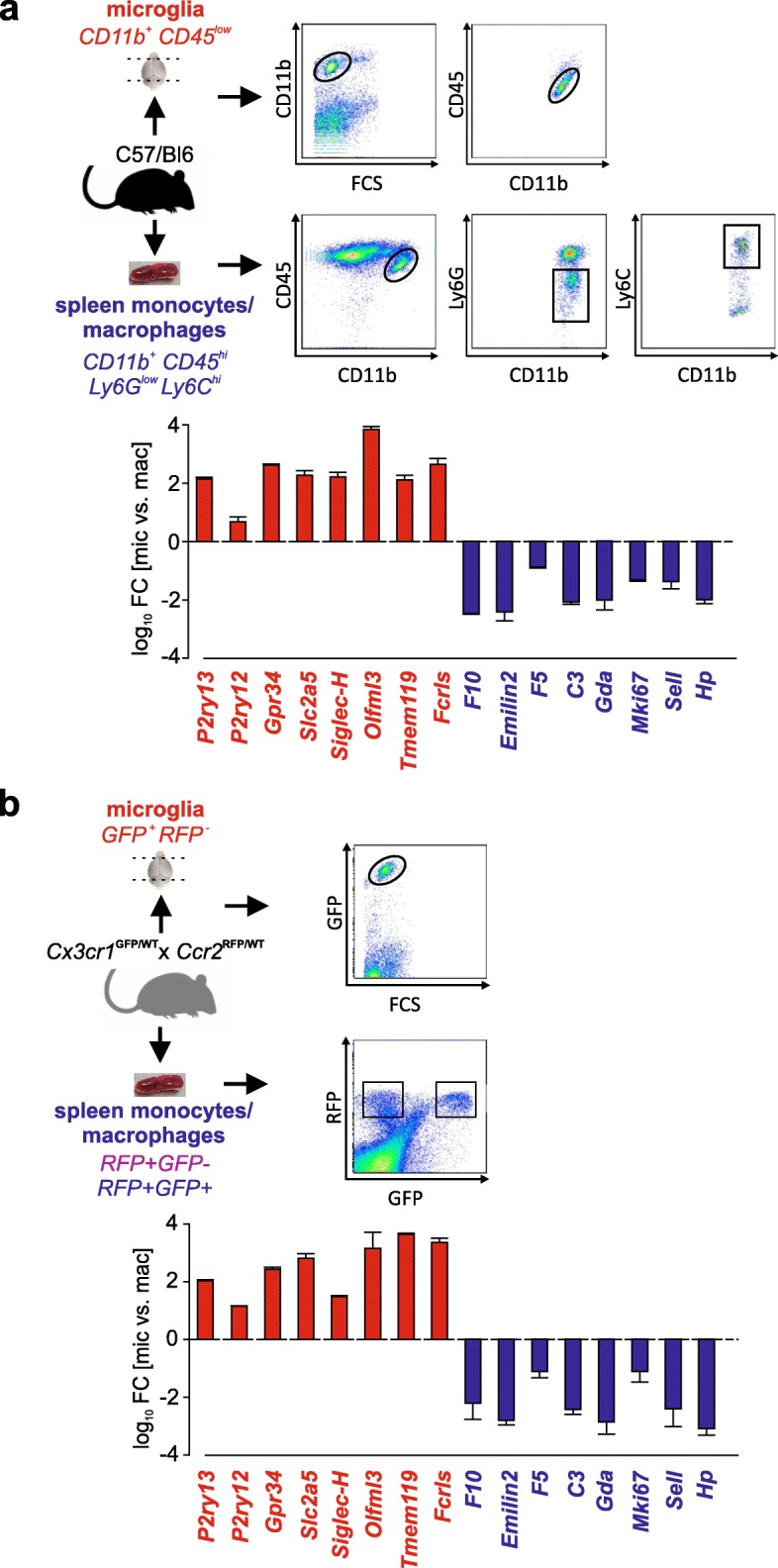
Bar graphs represent the log_10_ fold change expression (panel **a** and **b**)

**Fig. 5:**
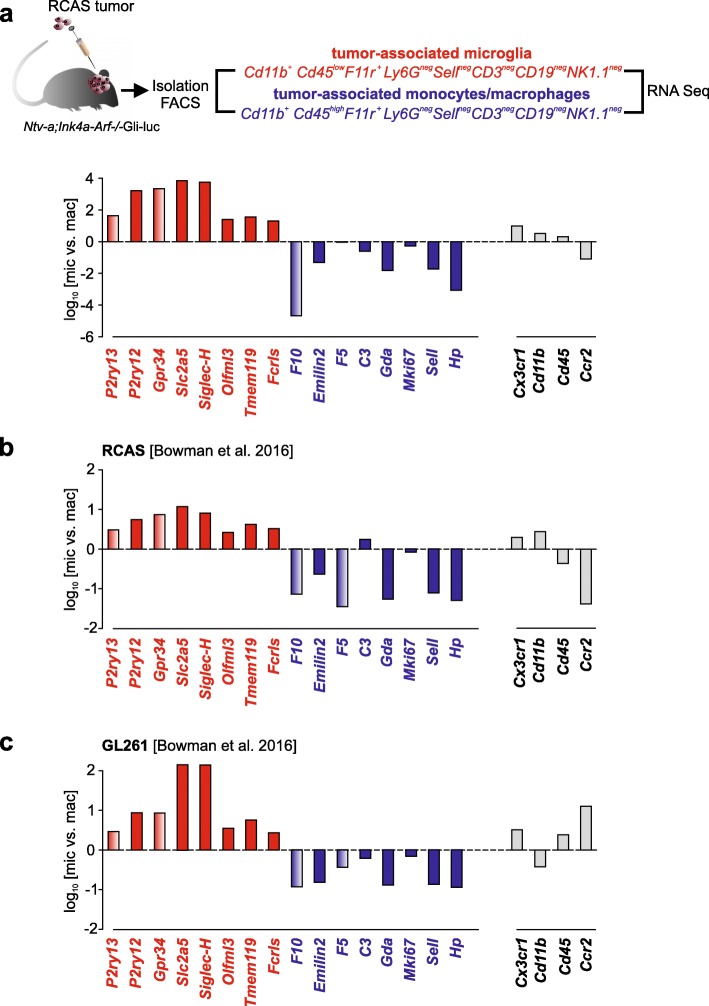
Bar graphs represent the log_10_ fold change expression (panel **a**, **b** and **c**)
