# Supplementary figures and images for: Comprehensive gene expression meta-analysis identifies signature genes that distinguish microglia from peripheral monocytes/macrophages in health and glioma
Source: Acta Neuropathol Commun. 2019 Feb 14;7:20. doi: 10.1186/s40478-019-0665-y (PMC6376799; doi:10.1186/s40478-019-0665-y)

**a**

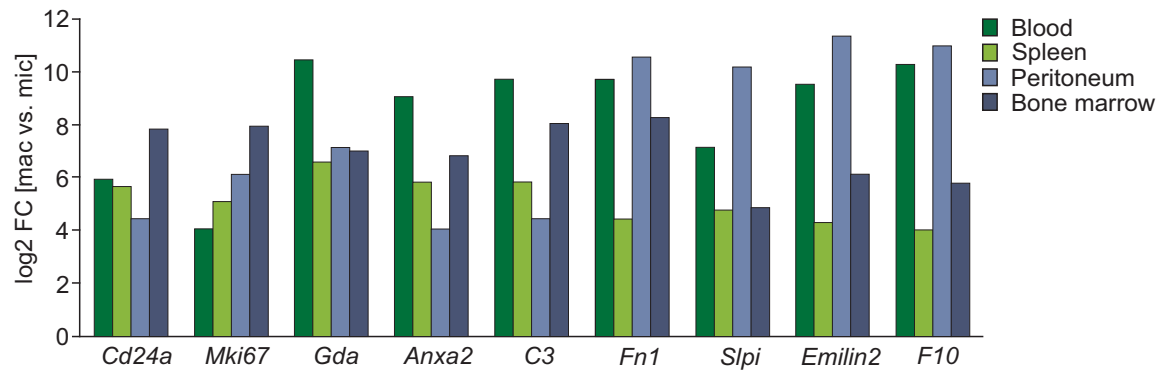

**b**

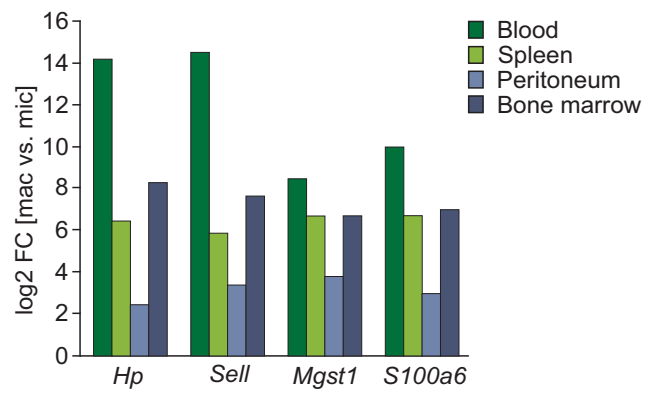

Supplement: Supplementary file 1 — Figure S1.. Expression levels of selected differentially expressed macrophage marker genes after hierarchical clustering in peripheral monocyte/macrophage subpopulations isolated from blood, spleen, peritoneum and bone marrow. (a) The differentially-expressed SGmac genes, which were identified in cluster 1 (Cd24, Mki67, Gda, Anxa2, C3, Fn1, Slpi, Emilin2, F10) following hierarchical clustering of the 145 significantly enriched and specific peripheral monocyte/macrophage genes shared across all five datasets, are shown. Expression is shown as the log2 fold change of expression of the peripheral monocyte/macrophage subpopulations isolated from blood (dark green; [5]), spleen (light green; [7]), peritoneum (light blue; [22]) and bone marrow (dark grey; [33]) compared to microglia for each of the datasets. For bone marrow-derived monocyte/macrophages, the RNA-sequencing dataset from Pong et al. is shown [33]. (b) The differentially-expressed SGmac genes, which were identified in cluster 2 (Hp, Sell, Mgst1 and S100a6) following hierarchical clustering of the 145 significantly enriched and specific peripheral monocyte/macrophage genes shared across all five datasets, are shown. Expression is shown as the log2 fold change of expression of the peripheral monocyte/macrophage subpopulations isolated from blood (dark green; [5]), spleen (light green; [7]), peritoneum (light blue; [22]) and bone marrow (dark grey; [33]) compared to microglia for each of the datasets. For bone marrow- derived monocyte/macrophages, the RNA-sequencing dataset from Pong et al. is shown [33]. (PDF 393 kb) [file 40478_2019_665_MOESM1_ESM.pdf]

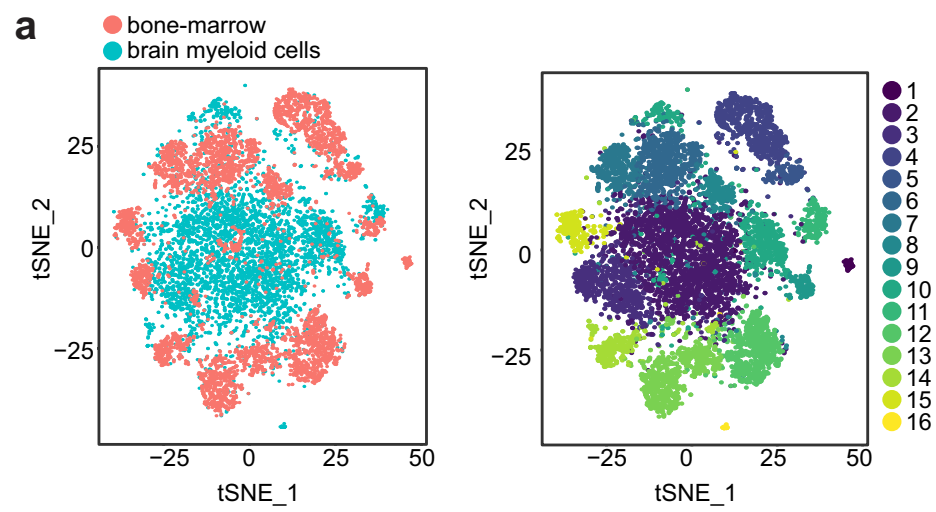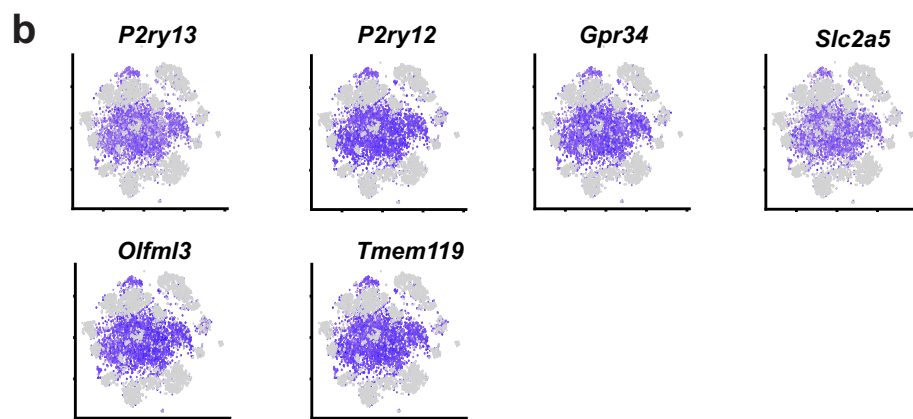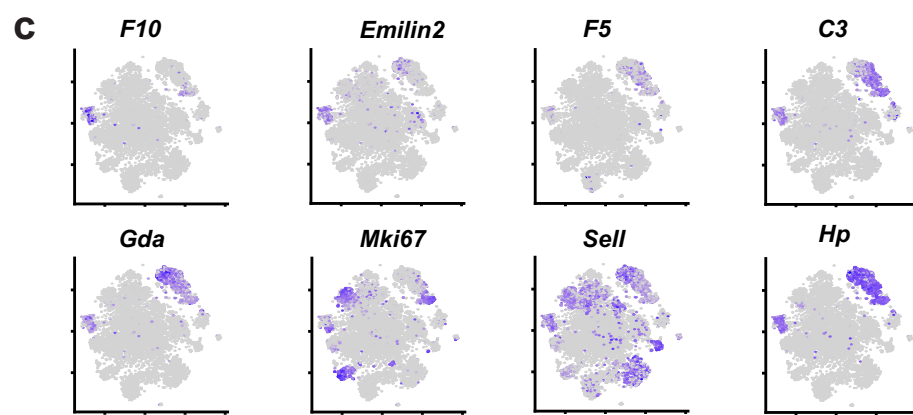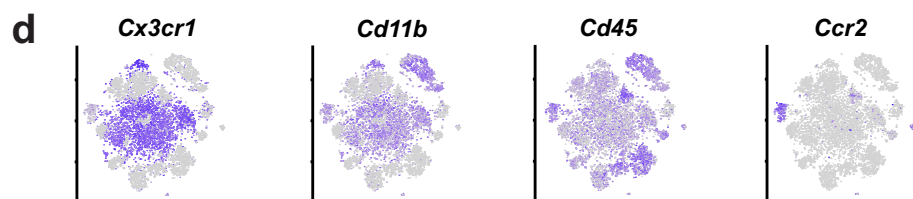

Supplement: Supplementary file 2 — Figure S2. Spatial visualization, clustering and expression of SGmic, SGmac and classical monocyte/macrophage marker genes in single cell sequencing data derived from brain myeloid and bone marrow cells. (a) t-distributed Stochastic Neighbor Embedding (t-SNE) spatial visualization and clustering of brain myeloid single cells (microglia; turquoise) dataset and bone marrow single cells (red) dataset derived from the single cell sequencing data of the Tabula Muris Consortium [42]. The right panel depicts clusters 1–16 represent all different cell populations detected by automatic clustering (Seurat FindCluster function). (b) t-SNEs showing the expression of the SGmic genes within the spatial distribution of brain myeloid and bone marrow cells. Identified SGmic genes from the analyzed single cell sequencing dataset comprise P2ry13, P2ry12, Gpr34, Slc2a5, Olfml3, Tmem119. (c) t-SNEs showing the expression of the SGmac genes (F10, Emilin2, F5, C3, Gda, Mki67, Sell, Hp) within the spatial distribution of brain myeloid and bone marrow cells. (d) t-SNEs showing the expression of the canonical monocyte/macrophage markers (Cx3Cr1, Cd11b, Cd45 and Ccr2) within the spatial distribution of brain myeloid and bone marrow cells. (PDF 14645 kb) [file 40478_2019_665_MOESM2_ESM.pdf]

**a**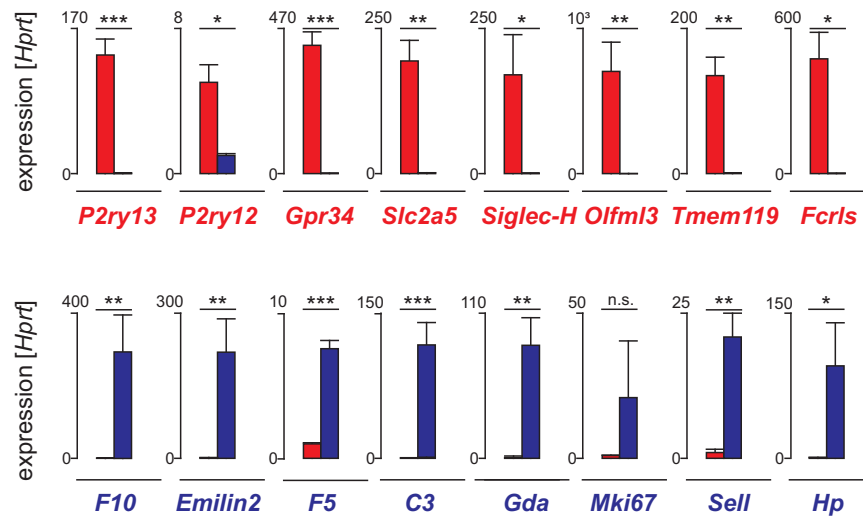**b**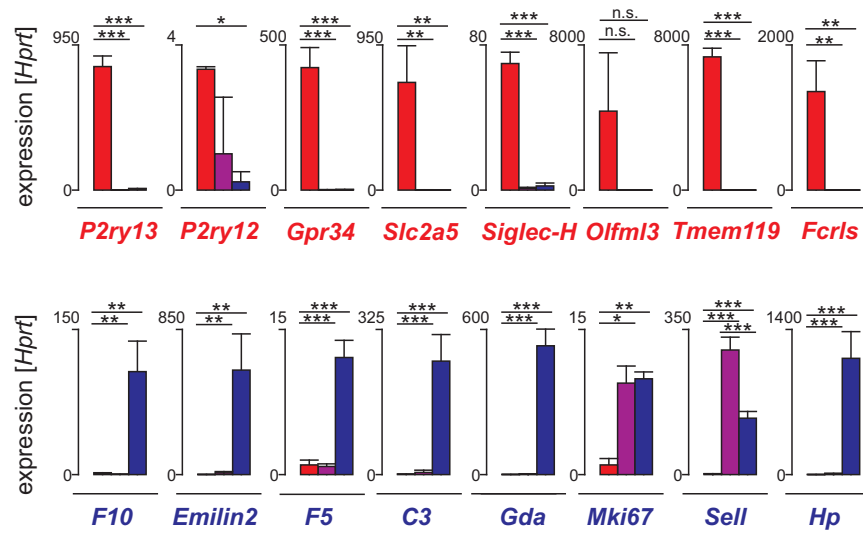

Supplement: Supplementary file 3 — Figure S3. Validation of SGmic and SGmac genes in microglia and peripheral monocytes/macrophages freshly isolated from two different mouse models. (a) Microglia (CD11b+ CD45low; red) and circulating spleen monocytes (CD11b+ CD45high Ly6Glow Ly6Chigh; blue) were freshly isolated from 12-week-old male C57/Bl6 WT mice by FACS and the expression of SGmic genes (P2ry13, P2ry12, Gpr34, Slc2a5, Siglech, Olfml3, Tmem119, and Fcrls; red) and SGmac genes (F10, Emilin2, F5, C3, Gda, Mki67, Sell, Hp; blue) determined by quantitative RT-PCR. Bar graphs represent the fold change expression of marker genes normalized to Hprt, where SGmic genes (red) are shown in relation to the spleen monocyte/macrophage population (CD11b+ CD45high Ly6Glow Ly6Chigh; blue) and SGmac genes (blue) compared to microglia (n = 3). For statistical analysis, unpaired t-tests were performed. * = P < 0.05; ** = P < 0.01; *** = P < 0.001. (b) Microglia and spleen monocytes/macrophages were freshly isolated from 8 to 12-week-old male Cx3cr1GFP/WT;Ccr2RFP/WT mice by FACS, GFP+RFP− cells representing microglia (red), RFP+GFP+ (blue) and RFP+GFP− (purple) cells representing spleen monocytes/macrophages. The expression of SGmic genes (P2ry13, P2ry12, Gpr34, Slc2a5, Siglech, Olfml3, Tmem119, and Fcrls; red) and SGmac genes (F10, Emilin2, F5, C3, Gda, Mki67, Sell, Hp; blue) was determined by quantitative RT-PCR. Bar graphs represent the fold change expression of each gene normalized to Hprt, where SGmic genes (red) are shown in relation to spleen monocytes/macrophages (RFP+GFP+; blue) and SGmac genes compared to microglia (GFP+RFP− cells; n = 3). For statistical analysis, one-way ANOVA following Bonferroni’s multiple comparison test was performed. * = P < 0.05; ** = P < 0.01; *** = P < 0.001. (PDF 401 kb) [file 40478_2019_665_MOESM3_ESM.pdf]

**a**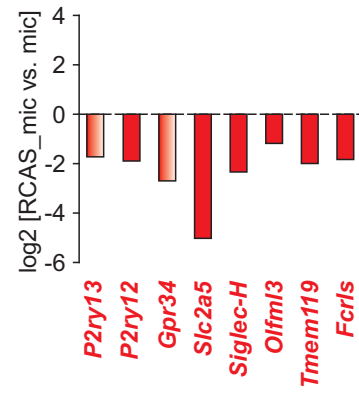**b**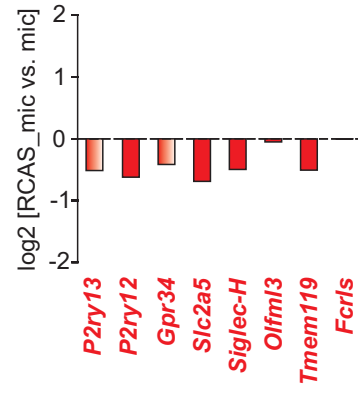**c**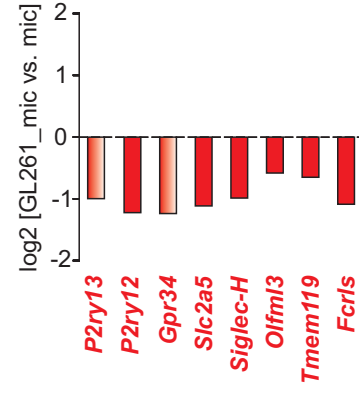

Supplement: Supplementary file 4 — Figure S4. Expression of SGmic genes in glioma-associated microglia as compared to healthy microglia in RNA sequencing datasets derived from healthy, RCAS glioma or GL261 glioma mice. (a) The log-fold change expression of SGmic genes (P2ry13, P2ry12, Gpr34, Slc2a5, Siglec-H, Olfml3, Tmem119, Fcrls) in glioma-associated microglia isolated from experimental RCAS tumors compared to microglia isolated from healthy control brains is shown. Expression data were extracted from RNA sequencing data generated by our group. (b) The log-fold change expression of SGmic genes (P2ry13, P2ry12, Gpr34, Slc2a5, Siglec-H, Olfml3, Tmem119, Fcrls) in glioma-associated microglia isolated from RCAS tumors compared to microglia isolated from healthy control brains is shown. Expression data were extracted from published RNA sequencing data [5]. (c) Graph shows the log-fold change expression of SGmic genes (P2ry13, P2ry12, Gpr34, Slc2a5, Siglec-H, Olfml3, Tmem119, Fcrls) in glioma-associated microglia isolated from GL261 tumors as compared to microglia isolated from healthy control brains. Expression data were extracted from published RNA sequencing data [5]. (PDF 394 kb) [file 40478_2019_665_MOESM4_ESM.pdf]
